# Statistical process monitoring to improve quality assurance of inpatient care

**DOI:** 10.1186/s12913-019-4866-7

**Published:** 2020-01-07

**Authors:** Lena Hubig, Nicholas Lack, Ulrich Mansmann

**Affiliations:** 1 Institute for Medical Information Processing, Biometry, and Epidemiology, Ludwig-Maximilians-Universität, Marchioninistr. 15, Munich, 81377 Germany; 2 Bavarian Institute for Quality Assurance, Munich, 80331 Germany

**Keywords:** Statistical process control, CUSUM, Hospital performance, Quality assurance

## Abstract

**Background:**

Statistical Process Monitoring (SPM) is not typically used in traditional quality assurance of inpatient care. While SPM allows a rapid detection of performance deficits, SPM results strongly depend on characteristics of the evaluated process. When using SPM to monitor inpatient care, in particular the hospital risk profile, hospital volume and properties of each monitored performance indicator (e.g. baseline failure probability) influence the results and must be taken into account to ensure a fair process evaluation. Here we study the use of CUSUM charts constructed for a predefined false alarm probability within a single process, i.e. a given hospital and performance indicator. We furthermore assess different monitoring schemes based on the resulting CUSUM chart and their dependence on the process characteristics.

**Methods:**

We conduct simulation studies in order to investigate alarm characteristics of the Bernoulli log-likelihood CUSUM chart for crude and risk-adjusted performance indicators, and illustrate CUSUM charts on performance data from the external quality assurance of hospitals in Bavaria, Germany.

**Results:**

Simulating CUSUM control limits for a false alarm probability allows to control the number of false alarms across different conditions and monitoring schemes. We gained better understanding of the effect of different factors on the alarm rates of CUSUM charts. We propose using simulations to assess the performance of implemented CUSUM charts.

**Conclusions:**

The presented results and example demonstrate the application of CUSUM charts for fair performance evaluation of inpatient care. We propose the simulation of CUSUM control limits while taking into account hospital and process characteristics.

## Background

Statistical Process Monitoring (SPM) as a means to monitor performance has become a popular method in the health care sector, for example in the monitoring of surgical outcomes or clinical performance [1–5]. Using SPM and in particular the cumulative sum (CUSUM) method applied here, it is for example possible to decide whether a recent change in personnel or hospital organisation has lead to a decline in quality or whether, on the contrary, the new surgical team or hospital reorganisation has improved the quality of care in the hospital.

In this contribution, we assess the use of CUSUM method in health care by considering the example of quality assurance of inpatient care and demonstrate the applicability and explain application details of the CUSUM in this context. The use of SPM in this area may greatly benefit the overall performance of the hospital, as processes are continuously monitored and process deviations are detected as soon as data are available, allowing for rapid interventions.

In our example, the hospitals reporting the monitored performance indicators vary greatly in their characteristics. Thus, to ensure fair comparison of hospitals, different cause of variance have to be considered. Adjusting control charts for different risk populations is in many cases available and has been discussed before [6]. Additionally, emphasis should be placed on the difference in number of patients treated by the hospitals in the same time frame. An important parameter to evaluate the performance of control charts is the probability of a false alarm of the control chart. A false alarm is the incorrect signal of a process change. False alarms should be avoided as best one can to enhance the trust in the method and the reliability of the alarms.

CUSUM charts are considered optimal for indicating small performance shifts, although their statistics are not directly interpretable [7–9]. Originally formulated by Page in 1954 [10], CUSUM charts have since been applied to non-risk-adjusted and risk-adjusted processes, which was described by Steiner et al. for the Bernoulli process [11]. Like many control charts, a change in performance of a monitored process is detected by two horizontal control limits, the upper limit indicating process deterioration and the lower limit process improvements. The setting of these control limits is crucial for the sensitivity of the CUSUM chart as they determine if a chart is able to detect deteriorations early on, has a considerable detection delay or overlooks them completely. We must stress that to our knowledge there are no closed analytical expressions for control limits in CUSUM charts which are applicable here. This implies that there may be multiple possible ways to choose those control limits, each with their own advantages and drawbacks.

One frequently considered method to construct control limits are based on selecting the average run length (ARL) of the CUSUM chart [11–13]. For this purpose an appropriate ARL is determined for the process at hand, and subsequently control limits are identified that yield this desired ARL. Markov-Chain-approximations were first described by Brook and Evans for approximating ARL of control charts [14]. Recently, Knoth et al. proposed a more exact and accurate Markov-Chain approach for estimating the ARL of risk-adjusted CUSUM charts [15]. Considering that the distribution of run lengths is skewed to the right, it remains difficult to deduce prior alarm rates of control charts when constructing a control scheme based on ARL, and it is therefore difficult for clinical practitioners to choose an appropriate ARL.

In this work, we suggest to instead use simulation of control limits for Bernoulli log-likelihood CUSUM charts based on the probability of a false alarm within a process. We study control limits determined by a false alarm probability and their dependence on specific features such as hospital volume, baseline failure probability and case risk mix for non-risk-adjusted and risk-adjusted performance indicators. We conduct simulation studies in order to investigate alarm characteristics of CUSUM designs to monitor hospital performance. Subsequently, we give an example based on hospital performance data in Bavaria, Germany.

### Motivating example

This work was motivated by the need for real-time detection of quality deficits in quality assurance of hospitals in Bavaria, Germany. External quality assurance (EQA) in Germany is regulated by the Directive on Measures concerning Quality Assurance in Hospitals [16]. According to this directive, each patient’s quality of treatment is reflected in a set of nationally standardized performance indicators. The raw case based performance data are transmitted to the regulatory agency by the end of February following the reporting year. The annual mean of the performance indicator is then compared to the national target, and, if relevant deviations are detected, appropriate interventions are initiated.

Consequently, there is a considerable time lag between the date of event, evaluation, and intervention; in some cases up to one and a half years. Moreover, the quality assurance process in use masks trends and seasonal effects by evaluating aggregated data.

The data set allows for early sequential analyses, as hospitals transmit their performance data throughout the year, and all observations are recorded with a date of documentation.

In most cases, however, the date of documentation does not equal date of treatment, and sometimes multiple patients are documented at the same time. Political efforts for earlier data documentation and thus sooner analysis have begun, and hospitals are encouraged to document and transmit their performance data as soon as possible and continuously throughout the year to allow for interim analyses.

The Institute for Quality Assurance and Transparency in Health Care (Institut für Qualitätssicherung und Transparenz im Gesundheitswesen, IQTIG) is the regulatory agency responsible for quality assurance on federal level. At state level (Bundesland), quality assurance is supported by state offices. In Bavaria, this is the Bavarian Agency for Quality Assurance (Bayerische Arbeitsgemeinschaft für Qualitätssicherung).

Three performance indicators were chosen to show the application of CUSUM in EQA (Table 1). The indicators are developed by the IQTIG, and the indicators’ specifications are published on the website of the IQTIG [17]. To illustrate the risk-adjusted CUSUM chart, we selected a performance indicator (11724), which reflects in-hospital complications or death after open carotid stenosis surgery. The risk model is published by the IQTIG and updated annually [18]. For 2016, the explanatory variables were: age, indication group, preoperative degree of disability and ASA classification. The hospital result is given as a rate of observed to expected cases. In the years 2016 and 2017, the average result of hospitals in Bavaria were 1.27 and 1.18 respectively.

**Table 1:** EQA performance indicators selected for simulation

Number	Type	Description	Result	Result
			(Bavaria, 2016)	(Bavaria, 2017)
51838	Crude	Neonatology: Surgically treated necrotizing enterocolitis in small premature infants	1.07*%*	1.47*%*
54030	Crude	Trauma surgery: Preoperative stay over 24 hours for patients with proximal femur fracture	20.35*%*	18.01*%*
11724	Risk-adjusted	Carotid Stenosis Surgery: Ratio of observed to expected cases of severe stroke or death under open surgery	1.27	1.18

The standard non-risk-adjusted CUSUM chart is illustrated by two crude performance indicators. Indicator (51838) represents a process with very low failure probability. It measures the events of surgically treated necrotizing enterocolitis in small premature infants, a serious intestinal infection often leading to death [19]. The average failure probability for this indicator increased from 1.07% to 1.47% in Bavarian hospitals from 2016 to 2017. Indicator (54030) measures rates of extended preoperative stay of patients with proximal femur fracture. Rapid surgery within 24 hours can prevent severe complications such as thrombosis, pulmonary embolism and pressure ulcers [20]. Bavarian hospitals slightly improved in this performance indicator from 2016 to 2017, as the failure probability decreased from 20.35% to 18.01%.

## Methods

### Cumulative Sum (CUSUM) chart

CUSUM charts for monitoring process performance for a deterioration in quality over time are defined as:[10]

1 $$ C_{t}=\text{max}(0, C_{t-1} + W_{t}), \quad t=1,2,3,...   $$

The dichotomous outcome of observation *y* equals 0 for every success and 1 for every adverse event. Observations are plotted in sequence of their temporal occurrence. Depending on the outcome, the CUSUM decreases or remains at zero for every success, and increases for every adverse event. The magnitudes of increase and decrease are denoted by CUSUM weights *W*_*t*_. Following Steiner et al. the weights *W*_*t*_ for the non-risk-adjusted CUSUM (ST-CUSUM) are:[11]

2 $$ W_{t}=\left\{\begin{array}{cc} \log{\left(\frac{1-c_{A}}{1-c_{0}}\right)} &\text{if}\ y_{t}=0\\ \log{\left(\frac{c_{A}}{c_{0}}\right)} &\text{if}\ y_{t}=1 \end{array}\right.,   $$

where *c*_0_ is the baseline failure probability and *c*_*A*_ the smallest unacceptable failure probability, which is the change in performance that is detected. CUSUM weights may be individualized for patient risk in the risk-adjusted CUSUM (RA-CUSUM). Here, the weights are:[11]

3 $$ W_{t}=\left\{\begin{array}{cc} \log{\left(\frac{1}{1-p_{t}+R_{A}p_{t}}\right)} &\text{if}\ y_{t}=0\\ \log{\left(\frac{R_{A}}{1-p_{t}+R_{A}p_{t}}\right)} &\text{if}\ y_{t}=1 \end{array}\right.,   $$

where *p*_*t*_ represents the individual patient risk score. The baseline failure probability is no longer constant, but tailored to patients’ risk. The risk-adjusted CUSUM monitors for a change in risk specified by an odds ratio change from *R*_0_ to *R*_*A*_, with *R*_*A*_ greater than one indicating process deteriorations.

### Factors influencing CUSUM chart performance

Several factors influence the performance and characteristics of the CUSUM performance and are considered in the simulation study. Some factors may be regarded as control switches of the monitoring schemes, as they are configurable and directly influence the control charts. Other factors are mostly fixed by the process that is monitored. Most of these factors are also relevant when applying other types of performance monitoring or SPM. Additionally, other types of variations exits that may influence the performance of CUSUM charts, but they are not accounted for. These may be unknown or random factors that are not measured or difficult to quantify, e.g. the quality of the data. Performance indicator: Performance indicators quantify a process output, indicating quality of care. For each performance indicator, the subset of patients covered by this indicator are specified. The performance indicator establishes the baseline failure probability *c*_0_ or the risk-adjustment model for the patients’ risk scores *p*_*t*_. Additionally, the performance indicator should be considered when setting up a monitoring scheme due to the implications of the process at hand on detecting performance deteriorations. Hospital volume: Hospital volume is here defined as the annual number of patients per performance indicator and hospital. It is a major source of variation between hospitals and possibly also within hospitals across years and is considered for fair performance evaluation in the control limit simulation as the sequence length *n*. As the hospital volume directly influences the control limit, it has a considerable effect on CUSUM performance. Case risk mix: Adjusting for individual patient risk is necessary when comparing outcomes, but there is often some uncertainty about the validity of the risk adjustment model. When possible, previous experience of the process can be used to estimate the case risk distribution (Phase I). This estimation of case risk mix is used in the simulation of the control limit, where outcome data is simulated on the estimated risk population. Detection level *δ*: Detectable changes in performance are determined by an odds ratio multiplier *δ*. In the ST-CUSUM, this change of *δ* defines the alternative failure probability *c*_*A*_, which influences the CUSUM weights *W*_*t*_ in Eq. 2. For the RA-CUSUM, *δ* is equal to *R*_*A*_ in Eq. 3. Values of *δ* greater than one detect process deteriorations, while values less than one detect process improvements. False alarm probability: We define the probability for a false alarm as the type 1 error of the CUSUM chart. It is the probability of a CUSUM signal within the monitoring of a process when the process is truly in control. Here it is applied as the defining parameter to construct CUSUM charts in the simulation of control limits.

### Defining the control limit

The CUSUM chart signals a process change when the CUSUM statistic exceeds a control limit. The process should then be investigated for quality deficits and monitoring can restart by resetting the current CUSUM statistic [21]. Control limits should be set after careful consideration of the probability of a false alarm and true alarm. As the alarm probabilities approach 100% with increasing run length, these parameters have to be estimated for a fixed sample size (*n*). For very small sample sizes it is possible to estimate the exact false alarm probability of possible control limits (Additional file 2). For larger sample sizes, we propose the following algorithm to select a control limit that will result in a specific false alarm probability:

1. Simulate a sufficiently large number of in-control sequential outcome data for *t*=1,2,…,*n*, with baseline failure probability or, if applicable, individual risk probabilities drawn from the population.
2. Unrestricted CUSUM runs are calculated for these simulated sequences. This means the CUSUM charts do not include a control limit and are not reset.
3. The maximum CUSUM statistics (*C*_*t*_) are collected from each CUSUM run.
4. The desired control limit for a sequence of size *n* is the (1−*P*(false alarm))-percentile of the maximum CUSUM statistics.

Software for computing CUSUM charts is available in an open source R package on the *Comprehensive R Archive Network (CRAN)*. The cusum package provides functions to simulate control limits for a false alarm probability, calculate CUSUM charts, and evaluate the true and false alarm probabilites of CUSUM charts [22]. Additional file 1 illustrates the construction of CUSUM charts using the cusum package for hospital performance data taking into account the previously described factors.

### Simulation study

**Fig. 1:**
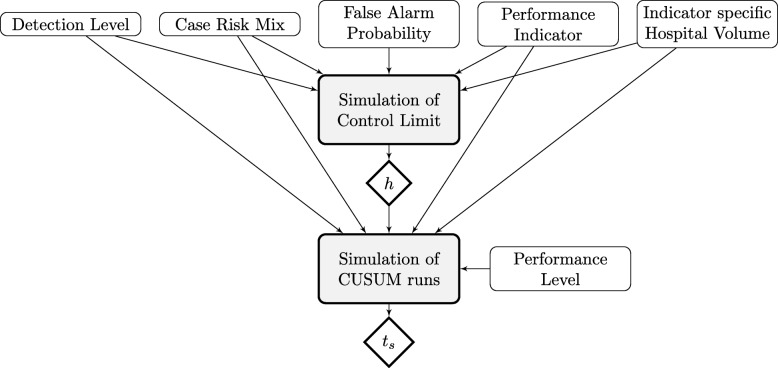
Simulation Plan. Factors influencing simulation of control limits (*h*) and time to signal (*t*_*s*_) in CUSUM runs simulation

We simulated hospital performance data to assess the effect of different influencing factors on the probabilities of false and true alarms of ST-CUSUM and RA-CUSUM charts. Figure 1 illustrates how the described factor influence the construction and simulation of CUSUM charts.

CUSUM runs are simulated for the three previously described IQTIG indicators from EQA. The baseline failure probabilities for the crude performance indicators were set to the national average failure rate of 2016 and 2017 (51838: *c*_0_=1.25*%*; 54030: *c*_0_=19.21*%*). For the risk-adjusted indicator 11724 we resampled risk scores with replacement from the total hospital population of 2016 and 2017. Additionally, we created artificial subpopulation based on case risk mix. For a high risk population risk scores were sampled from the risk population of the upper 25th percentile (≥ 1.04*%*). A low risk population was considered with risk scores sampled from the risk population of the lower 25th percentile (≤ 0.56*%*).

Three hospital volumes were derived for small, medium and large hospitals. The volume was estimated by taking the mean of the hospital volume percentiles across all performance indicators. The mean of hospitals below the 25th percentile (*n*_*s*_=7) was used as an estimate for small hospitals, the mean between the 25th and 75th percentile (*n*_*m*_=42) for medium hospitals, and the mean above the 75th percentile (*n*_*l*_=105) for large hospitals.

100 000 CUSUM runs were simulated to estimate control limits based on a false alarm probability. We simulated control limits for false alarm probabilites of 0.1%, 0.5%, 1% and 5%, in accordance with typical values of type 1 error rates. The CUSUM was set to detect deteriorations with *δ*> 1. The detection level of a doubling (2) of odds was considered as well as one step below (1.5) and one (2.5) and two (3) steps above.

To assess how well the specific CUSUM chart differentiates between good and poor performance, 2000 CUSUM runs were simulated for each control limit for in- and out-of-control performance. From these runs we collected the run length to signal, where the CUSUM statistic first exceeds the control limit. Finally, the signal rates are calculated as the proportion of CUSUM runs that are shorter than the hospital volume.

## Results

### Simulation results

For every hospital volume, performance indicator, and risk population, sixteen control limits were simulated for varying false alarm probabilities and detection levels.

Control limits are wider when the false alarm probability is small, detection level is high, baseline failure probability or case risk mix is high and hospital volume is large. This pattern was generally reflected in the simulated control limits.

**Fig. 2:**
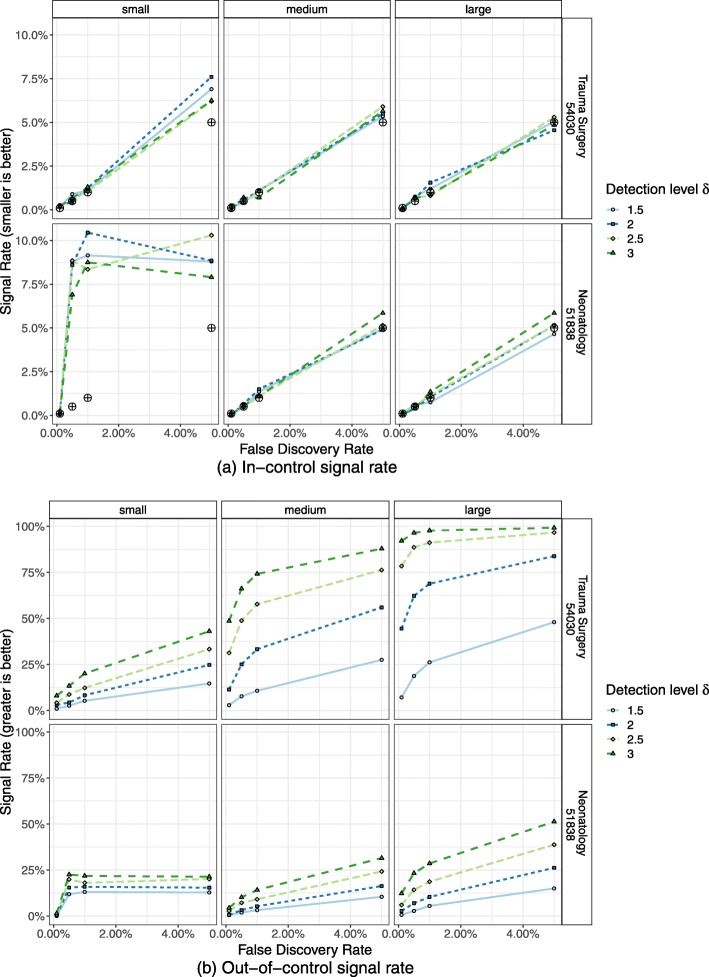
Simulation of ST-CUSUM. Percentage of ST-CUSUM charts signalling a process deterioration (alarm rate) from 2000 simulated in-control (top) and out-of-control (bottom) ST-CUSUM runs. The desired false alarm probability is marked by black symbols. **a** In-control signal rate. **b** Out-of-control signal rate

**Fig. 3:**
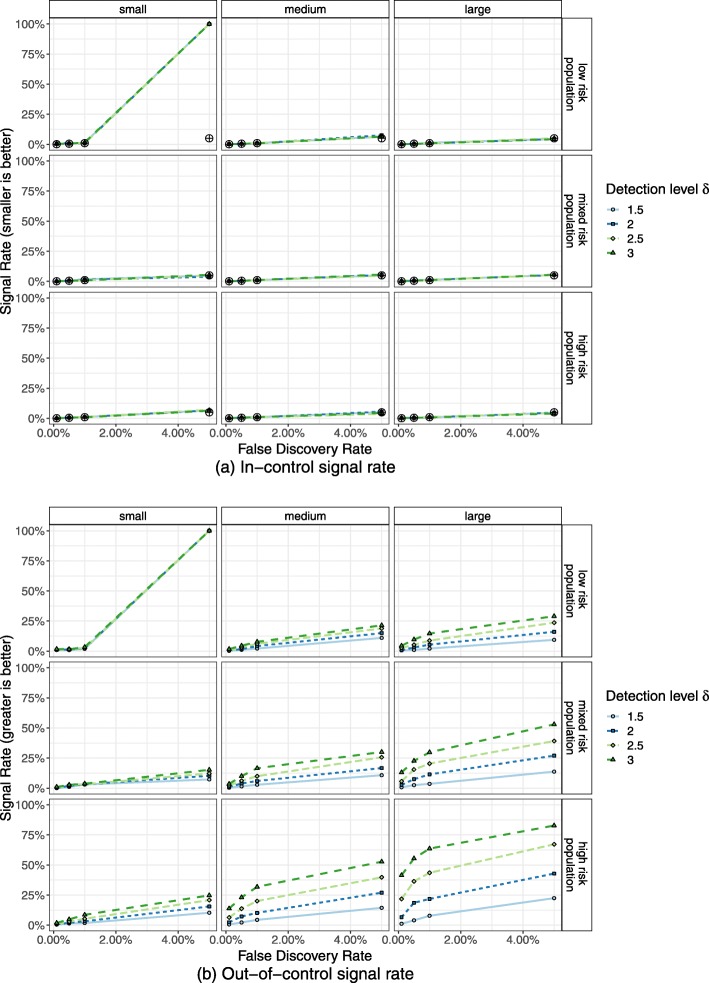
Simulation of RA-CUSUM. Percentage of RA-CUSUM charts signalling a process deterioration (alarm rate) from 2000 simulated in-control (top) and out-of-control (bottom) for risk-adjusted indicator 11724. RA-CUSUM runs were simulated for mixed, low and high risk populations. The desired false alarm probability is marked by black symbols. **a** In-control signal rate. **b** Out-of-control signal rate

In Figs. 2a and 3a the percentage of in-control CUSUM runs that signalled a process change are presented as signal rates. Here, performance was as expected and thus the signal rates should not exceed the predefined false alarm probability of the control limit.

The signal rates of in-control simulations were for the most part close to the desired false alarm probability, demonstrating successful simulation of control limits to obtain this false alarm probability. Only two populations of small hospital volume showed deviations from the desired false alarm probability and the observed in-control signal rate. For these scenarios, tight control limits had to be simulated, but due to the discrete nature of the CUSUM there are finite possible CUSUM control limits. For small hospital volume of indicator 51838 (Fig. 2a, bottom right), this was the CUSUM weight of an adverse event, which results in a higher false signal rate of ≈15*%*. As the risk-adjusted CUSUM individually weights adverse events based on the patient population, the control limit chosen for small hospital volume and low risk population of indicator 11724 (Fig. 3a, top left) was zero. This results in a CUSUM signal at every observation, reflected by the 100% in-control and out-of-control signal rates. For these scenarios it may be reasonable to choose a lower false alarm probability and in turn also accepting a lower power.

Signal rates for out-of-control CUSUM runs (Figs. 2b, 3b) represent the correctly identified deteriorations and ideally should be close to 100%.

Large hospital volumes and higher failure probability resulted in a higher power. Control chart of indicator 54030 achieved 99.25% for the highest false alarm probability and detection level.

Yet, most CUSUM runs had low power; particularly CUSUM runs for small hospital volumes did not trigger an alarm in the majority of CUSUM runs within one observation period.

### Application to EQA hospital performance data

CUSUM charts are applied to real data from EQA of inpatient care from the years 2016 and 2017 provided by the Bavarian Agency of Quality Assurance. Performance data from 2016 is used to estimate baseline failure probability and case risk mix (Phase I) to construct CUSUM charts for performance data of 2017 (Phase II), though the monitoring period extends from March 1st 2017 to February 28th 2018. This is because documentation and transmission deadline is February 28th for the previous year with the reporting year shifted by two months.

**Fig. 4:**
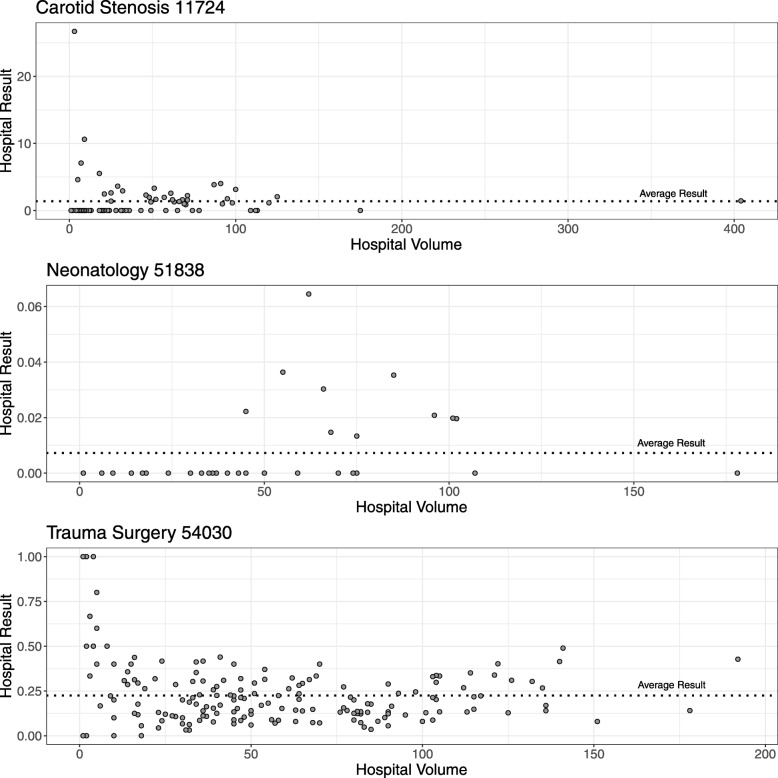
Hospital Results. Annual hospital results of performance indicators in Bavaria 2016

The hospital results and hospital volumes of the year 2016 are displayed in Fig. 4. Average hospital volume decreased from 2016 to 2017 from 50 observations to 48 observations.

Baseline failure probabilities for the two crude indicators are derived from the overall 2016 average (54030: *c*_0_=20.35*%*; 51838: *c*_0_=1.07*%*). Case risk mix for the risk-adjusted indicator was inferred from the 2016 hospital specific population. Patient individual risk for complications or death ranged between 0.24% to 40.98%, with a median of 0.83% across both years.

CUSUM charts were constructed by simulating the control limit for a false alarm probability of 5%. We set the detection level to *δ*=2 and constructed control charts for hospitals with hospital with more than one observation in 2016 and 2017. Indicator 54030 covers 163 hospitals, indicator 51838 45 hospitals and indicator 11724 64 hospitals.

We initiated all CUSUM runs with *C*_0_=0 and reset *C*_*t*_ to zero after every alarm, which is applicable if an investigation after an alarm takes place and appropriately identifies any problems [21].

Of the 261 hospitals’ CUSUM charts, 34 processes triggered an alarm and were identified as out-of-control. Overall, 86.21% of the hospitals were classified as in-control (Table 2). Out-of-control processes of indicators 51838 and 11724 had at most one alarm, and for indicator 54030 seven hospitals had more than one alarm.

**Table 2:** Percentage of hospitals with CUSUM alarms per performance indicator in Bavaria 2017

	54030	51838	11724
Alarms	(*n*=163)	(*n*=34)	(*n*=64)
0	85.89%	85.29%	88.00%
1	9.82%	14.71%	9.00%
2	1.84%	0.00%	0.00%
3+	2.45%	0.00%	0.00%
NA	0.00%	0.00%	3.00%

Two of the control charts for indicator 11724 had to be discarded due to incorrect control limit (Alarms: NA)

**Fig. 5:**
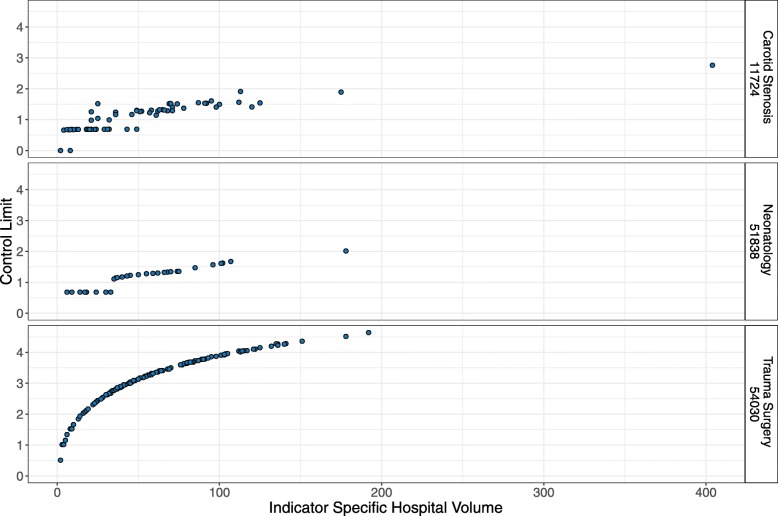
EQA Application. Control limits for hospital performance data of EQA in Bavaria. Control limits were estimated on performance data of 2016 and simulated for *δ* = 2 and a false alarm probability of 5%

**Fig. 6:**
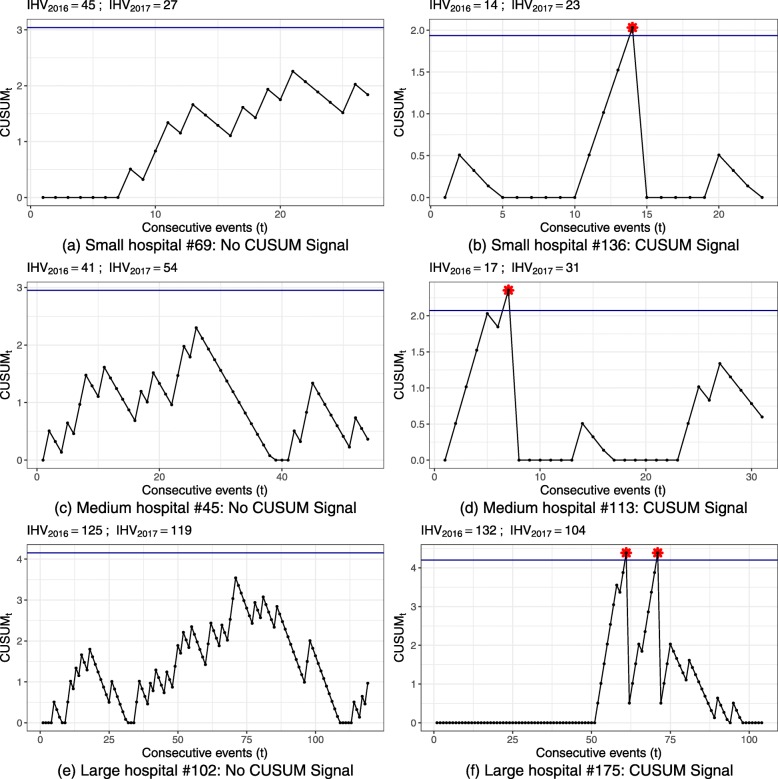
EQA Application Trauma Surgery 54030. Selected CUSUM plots for individual hospital annual performance data of 2017. **a** Small hospital #69: No CUSUM Signal. **b** Small hospital #136: CUSUM Signal. **c** Medium hospital #45: No CUSUM Signal. **d** Medium hospital #113: CUSUM Signal. **e** Large hospital #102: No CUSUM Signal. **f** Large hospital #175: CUSUM Signal IHV denotes indicator specific hospital volume.

**Fig. 7:**
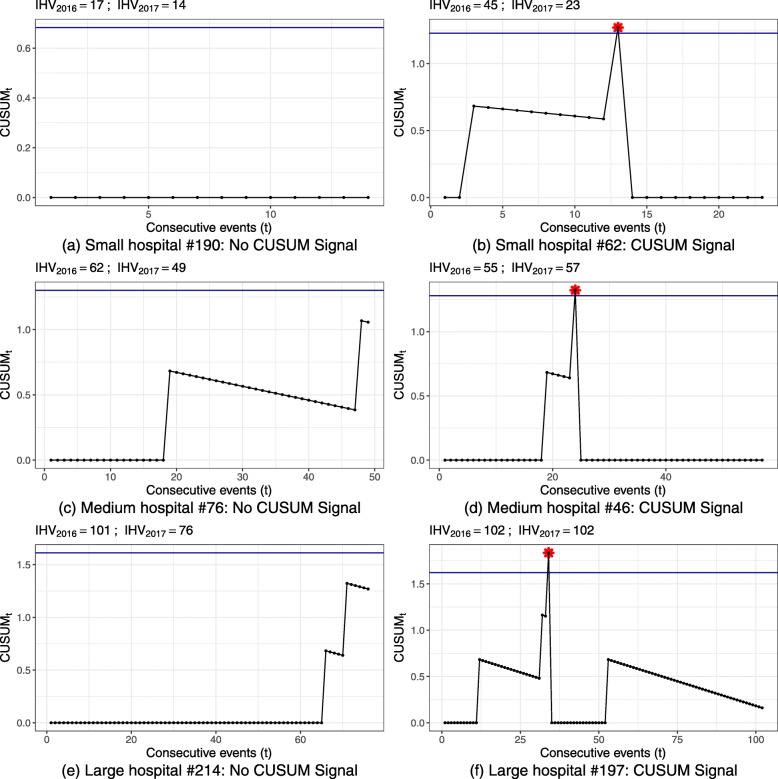
EQA Application Neonatology 51838. Selected CUSUM plots for individual hospital annual performance data of 2017. **a** Small hospital #190: No CUSUM Signal. **b** Small hospital #62: CUSUM Signal. **c** Medium hospital #76: No CUSUM Signal. **d** Medium hospital #46: CUSUM Signal. **e** Large hospital #214: No CUSUM Signal. **f** Large hospital #197: CUSUM Signal IHV denotes indicator specific hospital volume.

**Fig. 8:**
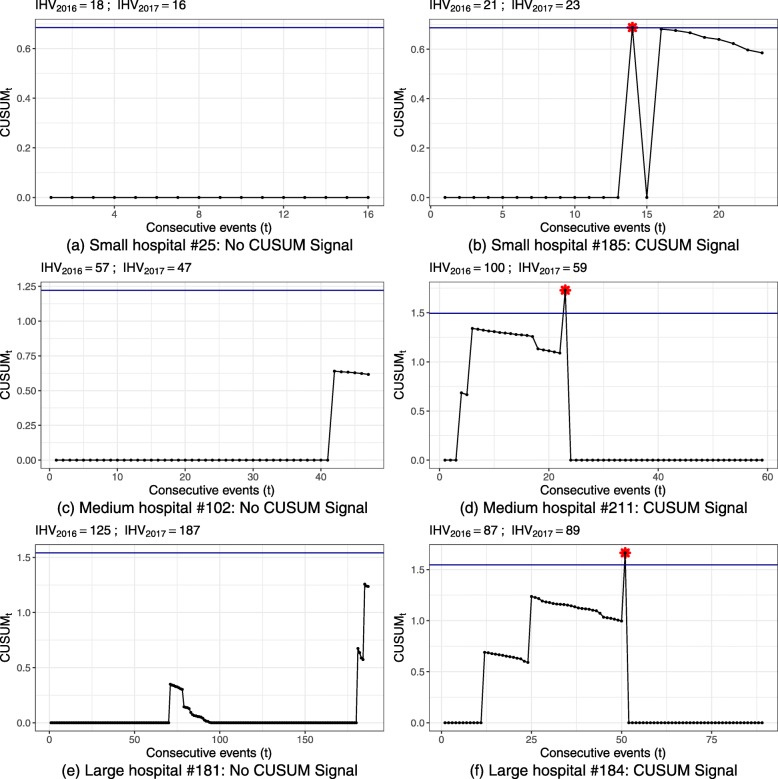
EQA Application Carotid Stenosis 11724. Selected CUSUM plots for individual hospital annual performance data of 2017. **a** Small hospital #25: No CUSUM Signal. **b** Small hospital #185: CUSUM Signal. **c** Medium hospital #102: No CUSUM Signal. **d** Medium hospital #211: CUSUM Signal. **e** Large hospital #181: No CUSUM Signal. **f** Large hospital #184: CUSUM Signal IHV denotes indicator specific hospital volume.

Figure 5 displays the resulting control limits for the CUSUM charts of all hospital processes. Exemplary CUSUM charts showing individual hospital processes are given in Figures 6 and 7 for the ST-CUSUM and Figure 8 for the RA-CUSUM. Simulated control limits of ST-CUSUM charts for indicators 54030 and 51838 increased with increasing hospital volume to ensure a constant false alarm probability during one observation period (Fig. 5). Control limits of the RA-CUSUM chart for indicator 11724 increased as well, but adjustment of the different case risk mixes influenced variability of the control limits. Similar to the simulation results, some control limits for smaller hospital volumes were estimated as the CUSUM weight for failure *W*_*t*_(*y*=1) or as zero. As the positive CUSUM weights *W*_*t*_(*y*=0), which decrease the CUSUM, were smaller for indicators 51838 and 11724 than for indicator 54030, adverse events were more difficult to compensate by good performance (Fig. 7b, Fig. 8f). CUSUM charts for indicators 51838 and 11724 categorized mostly only hospitals with less than two adverse events as in-control. The charts for indicator 54030 allowed for more adverse events, and also signalled multiple deteriorations. Hospitals with multiple alarms possibly had a persistent quality deficit in this indicator and were not able to control the process during the entire monitoring period. Some hospitals showed an accumulation of adverse events at specific points, which may help to locate causal deficits at subsequent investigation (Fig. 6f).

For larger hospital volume the wider control limits also allowed for more adverse events within a year. The large hospital #102 (Fig. 6e) was categorised as in-control for indicator 54030, although a third of the observations were adverse events. Hospital #113 (Fig. 6d) had 29% adverse events for indicator 54030 and triggered an alarm. This is partly due to the shorter sequence of adverse events and the smaller hospital volume. However, this hospital also had a substantial increase in volume from 2016 to 2017, so that the control limit probably was lower than necessary.

## Discussion

In this work we proposed the construction of CUSUM charts by simulating control limits for a predefined false alarm probability, and showed the alarm characteristics of ST- and RA-CUSUM charts regarding true and false alarm probability for different monitoring schemes and processes. The method of constructing control charts is intuitive and flexible regarding hospital volume and case risk mix.

### Strengths and limitations of this study

The control of false alarms in our method worked well for sufficiently large hospital volumes and high baseline failure probability. For very small sample sizes we presented an exact calculation of possible control limits and corresponding false alarm probabilities (Additional file 2). In monitoring schemes of small hospital volumes, it often remains impossible to adjust the control limit to fit a specific false alarm probability, as these control charts are not as flexible as control charts for larger volumes. Small hospitals continue to present an issue in SPM, as corresponding CUSUM charts are difficult to construct and evaluate. In our simulation, it is quite possible that no failure was simulated for small hospital volume processes (*n*_*s*_=7), especially for indicators with a small failure probability such as for indicator 51838 (*c*_0_=1.25*%*). Detecting a doubling or tripling of odds with a small failure probability and small hospital volume is difficult, as even with doubled or tripled odds, the probability to observe no adverse event is still large. Taking this example, 92% of *n*_*s*_=7 observations show no adverse events at failure probability *c*_0_ compared to 84% at doubled odds – i.e., in 84% of all possible sets of *n*_*s*_=7 patients, no difference between the in-control and out-of-control state is observable. As most control charts required at least two adverse events to signal, alarms became very unlikely. The hospitals’ CUSUM charts in the example showed that small hospitals may still benefit from an individual investigation based on the CUSUM chart as differences in performance are fairly well illustrated. Hospital volume may be increased by extending the data to cover multiple years, if the achievable false alarm probability is not acceptable.

The simulation study showed different results for processes with high baseline failure probability compared to those with low baseline failure probability. Again, this may be due to the simulation process, as low failure probability lead to few observable adverse events even when the process is out of control. Although simulation of out-of-control performance did not result in satisfactory power, the CUSUM did signal in the example and detected quality deficits in a similar rate as the processes with a high failure probability (Table 2). Current German regulations require that in cases of extremely adverse clinical outcome written explanations have to be furnished by the medical staff in every such instance. This strategy does not rule out the use of control charts for indicators with low baseline failure probability, and we suggest that individual investigations of adverse events should accompany CUSUM charts for these indicators. The monitoring of rare events is a common issue in SPM and Woodall and Driscoll gave a comprehensive review on this topic [23]. In this context, our example (*c*_0_=1.25*%*) is not yet regarded as rare, as the methods discussed here consider failure probabilities that are ten or a hundred times smaller.

As CUSUM charts are based on performance data of the previous year, they may be subject to uncertainty of these estimations. Monitoring across different years presents the additional challenge that specifications of performance indicators may change due to clinical recommendations of national advisory panels, and thus indicators may not always be comparable across different monitoring periods. Additionally, hospital volume and case risk mix vary across years, which affect the alarm characteristics of the CUSUM scheme. It has been shown that wrong expectations of risk mix or wrong model specifications can have a significant impact on CUSUM runs [13, 15, 24]. Zhang and Woodall proposed Dynamic Probability Control Limits (DPCL) for the CUSUM chart to address these issues [25]. These limits control the false alarm probability during monitoring by changing and updating the control limit based on new observations, but are more difficult to construct and interpret.

### Implications for policy and research

Augmenting established EQA with concurrent SPM may well help to improve the timeliness and the informative value of quality assurance. Quality deficits will be detected sooner than with analyses of aggregated means on an annual or quarterly basis. Thus, performance may be improved before any deteriorations are identified using conventional EQA. Moreover, adverse events are presented in their temporal context and trends or seasonal effects are more apparent in CUSUM charts. CUSUM charts can also be of assistance in the evaluation of intervention and will facilitate showcasing best practice examples.

Typically, there has to be a trade-off between low false alarm and high true alarm probability. Prioritizing a low false alarm probability will protect hospitals with good quality of care from false accusations. As all alarms require investigation at hospital level, false alarms will result in unnecessary draining of resources of monitoring investigators as well as of those investigated. Still, detecting deteriorations should not be disregarded and an adequate balance between false and true alarm probability should be sought out. A false alarm probability of 5%, which can result in an acceptable power, may be a reasonable choice for most scenarios.

In the example, we reset the CUSUM after every alarm to gain a sense of frequency of alarms. However, according to the theoretical background of SPM in industrial process control, this is only appropriate if the process is investigated and brought back in control, which is difficult to ensure in hospitals. Additionally, when the CUSUM restarts with the same control limit as before, the false alarm probability and power may be lower than anticipated, as the hospital volume decreases. If resetting the CUSUM to zero is not reasonable, resetting it to a greater value below the control limit is also an option. This was already proposed by Lucas and Crosier in 1982 [26], and results in faster subsequent alarms.

Use of risk-adjusted performance indicators should be further encouraged. Adjustment for case risk mix is necessary for a fair and robust quality assurance. If a risk-model for the particular indicator exists, risk-adjusted CUSUM charts are easy to implement and their performance in our study was similar to the standard CUSUM charts.

The problem of unknown temporal order of observation and its implication on CUSUM charts are of interest to explore further. For accurate CUSUM charts and for immediate intervention, observations should be recorded automatically with a precise time stamp. So far, the date of documentation remains an unsatisfactory surrogate parameter in place of date of treatment. Thus, hospitals that do not fulfil regular data documentation would have to be excluded from CUSUM analysis. In the future, even further advances in processing electronic health records can help to approximate real time bed-side performance evaluation. However, for the time being performance monitoring is still constrained by unnecessarily complicated and laborious processes of data documentation, transmission, validation and evaluation. Further advances in timely data documentation can be motivated by the prospect of implementing efficient SPM.

Benefits and issues arising from simultaneously monitoring multiple data streams should be dealt with more thoroughly when implementing CUSUM in German EQA. Multiple indicators of one hospital can provide additional information about the hospital’s performance. The global false discovery rate can, however, increase with multiple data streams, whether these are multiple indicators per hospital or multiple hospitals in the quality assurance. Previous work introduced controlling the false discovery rate by applying strategies from multiple testing to normally distributed data [27–29], and Mei proposed a scalable global monitoring scheme for concurrent data streams [30]. Methods to control the false discovery rate of multiple data streams need to be evaluated for their suitability in the monitoring scheme of German EQA.

## Conclusion

We propose a determination of control limits in CUSUM charts based on the false alarm probability and numerical simulations with appropriate adaptations to hospital volume and case risk mix. Exemplary resulting CUSUM charts are analysed with respect to their effective true and false alarm probabilities and pivotal CUSUM factors are pointed out. We demonstrate the feasibility of hospital volume and case risk mix adapted CUSUM charts in the external quality assurance of inpatient care.

## Supplementary information

**Additional file 1** Construct CUSUM charts for hospital performance. A vignette from the cusum R-package showcasing the construction of CUSUM charts for hospital performance data.

**Additional file 2** Exact Control Limits for small sample sizes. Calculation of exact control limits for small volumes and simulation result comparing the exact method to control limits simulated for a false alarm probability.

## Data Availability

The datasets generated during the simulation study are available from the corresponding author. Hospital performance data are not publicly available due to contracts restricting data access exclusively to explicitly named institutions conducting quality assurance for hospitals.

## Abbreviations

ARL: Average run length
CUSUM: Cumulative sum
EQA: External quality assurance
IQTIG: Institute for quality assurance and transparency in health care
RA-CUSUM: Risk-adjusted CUSUM
SPM: Statistical process monitoring
IHV: Indicator specific Hospital Volume
ST-CUSUM: Standard CUSUM (non-risk-adjusted)
