# Supplementary material for: Statistical process monitoring to improve quality assurance of inpatient care
Source: BMC Health Serv Res. 2020 Jan 7;20:21. doi: 10.1186/s12913-019-4866-7 (PMC6947979; doi:10.1186/s12913-019-4866-7)

Exact Control Limits for small sample sizes

Simulation Parameters

```
# sample sizes for control limit estimation
fap <- c(0.05, 0.01)
npat <- c(2:10)

# baseline failure probability
p0 <- seq(0.01, 0.2, 0.01)

# smallest rejected failure probability
o0 <- p0 / (1 - p0)
o1 <- o0 * 2
p1 <- o1 / (1 + o1)
```

Exact Control Limits

Exact control limits can be estimated with the following function:

```
small_hospital_boundary <- function(npat,
                                    failure_prob,
                                    pA) {
  #
  # This function calculates the exact distribution of the CUSUM
  # for a hospital with npat patients, the in control failure probability failure_prob
  # and the smallest inacceptable failure probability pA
  #

  outcome <- make_all_outcomes(npat_outcome = npat)

  p_failure <- apply(outcome, 1, function(kk, pp) {
    return(prod(ifelse(kk == 1, pp, 1 - pp)))
  }, pp = failure_prob)


  cs <- apply(outcome, 1, calc_cusum, c0 = failure_prob, cA = pA)
  cs_value <- unique(as.vector(cs))
  which.res <- lapply(cs_value, which.rfc, mm = cs)

  cs_distr <- lapply(which.res, function(yy, pp) {
    return(sum(pp[yy]))
  }, pp = p_failure)

  res <- cbind(cs_value, unlist(cs_distr))
  res <- res[sort.list(res[, 1]), ]
  colnames(res) <- c("cs_value", "fap_exact")
  return(res)
}
```

```
make_all_outcomes <- function(npat_outcome) {

  #
  #     This function creates all possible sequences of outcomes
  #

  m <- matrix(0:1, ncol = 1)

  for (ii in 2:npat_outcome) {
    m <- cbind(rbind(m, m), rep(0:1, c(1, 1) * 2^(ii - 1)))
  }

  return(m)
}

calc_cusum <- function(x, c0, cA) {

  #
  #     This function calculates the CUSUM chart
  #     for the given sequence of successes and failures
  #     provided by the vector x: x=0 no failure, x=1 failure
  #

  wt <- ifelse(x == 0, log((1 - cA) / (1 - c0)), log(cA / c0))

  j <- length(wt)
  ct <- rep(NA, j)
  ct[1] <- max(c(0, wt[1]))

  for (ii in 2:j) {
    ct[ii] <- max(c(0, ct[ii - 1] + wt[ii]))
  }
  return(ct)
}

which.rfc <- function(xx, mm) {
  res <- apply(mm, 2, function(yy, cc) {
    return(sum(yy >= cc) > 0)
  }, cc = xx)

  return(res)
}
```

The exact CL is selected by the closest possible false alarm probability to the desired false alarm probability. The remaining difference is shown in the next figure. Here, higher false alarm probability result in a higher error rate.

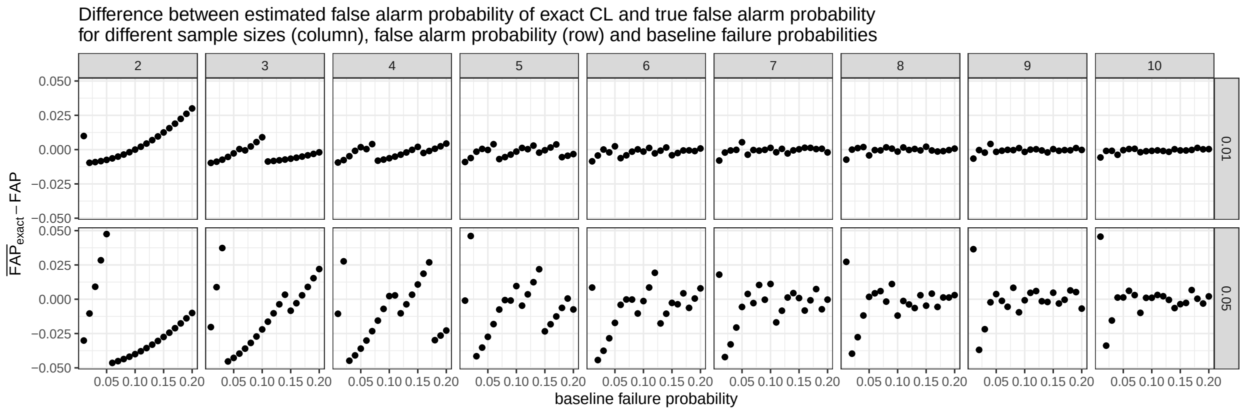

false alarm probability-simulated control limits

false alarm probability-simulated control limits are estimated by `cusum::cusum_limit_sim`. The simulation approaches the desired false alarm probability more conservatively.

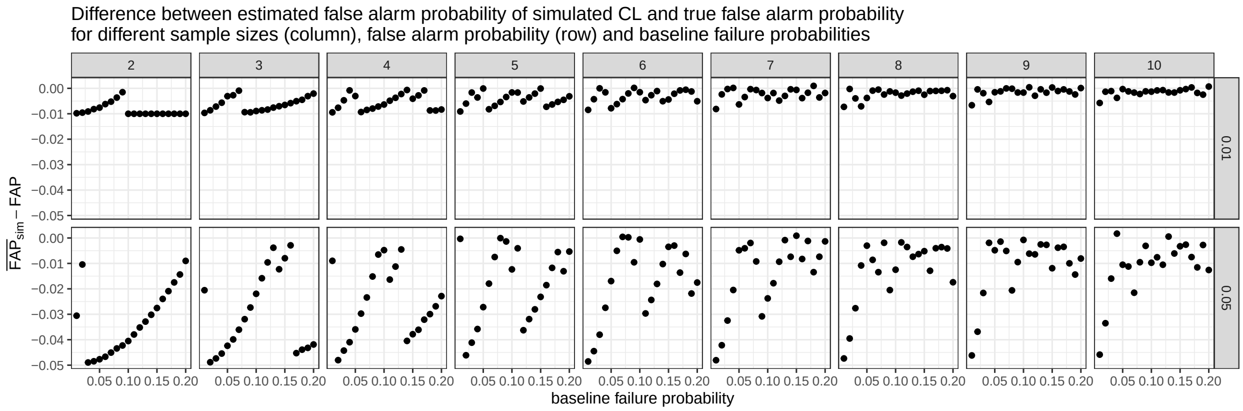

Difference between exact and false alarm probability-simulated control limits

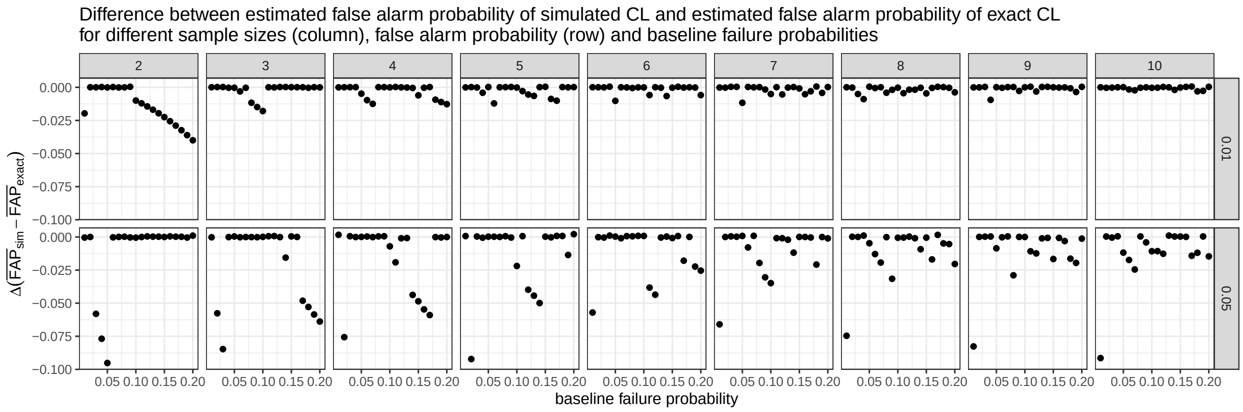

Supplement: Supplementary file 1 — Additional file 1 Construct CUSUM charts for hospital performance. A vignette from the cusum R-package showcasing the construction of CUSUM charts for hospital performance data. [file 12913_2019_4866_MOESM1_ESM.pdf]
