# Supplementary material for: Statistical process monitoring to improve quality assurance of inpatient care
Source: BMC Health Serv Res. 2020 Jan 7;20:21. doi: 10.1186/s12913-019-4866-7 (PMC6947979; doi:10.1186/s12913-019-4866-7)
Supplement: Supplementary file 2 — Additional file 2 Exact Control Limits for small sample sizes. Calculation of exact control limits for small volumes and simulation result comparing the exact method to control limits simulated for a false alarm probability. [file 12913_2019_4866_MOESM2_ESM.pdf]

Construct CUSUM charts for hospital performance

Overview

This vignette describes CUSUM charts based on a simulated false alarm probability for hospital performance data in the R package **cusum**. This is a practical guide to constructing and evaluating non-risk-adjusted and risk-adjusted CUSUM charts following Steiner et al. (Biostatistics 1.4 (2000), pp. 441-52).

The **cusum** packages takes different factors into account that influence the alarm rate of CUSUM charts. Some are given by the process to be monitored; these factors are:

- number of patients: How many observations do we expect in a monitoring period (e.g. a month/a year)?
- risk-adjustment: Is risk adjustment available? Can we allocate different risks to different observations?
  - if yes:
    - patient risks: What are these risks and how are they distributed?
  - if no:
    - accepted failure probability: What kind of failure rate do we expect on average?

The primary control input when constructing a CUSUM chart is the control limit. The control limit alarms performance deterioration once crossed by the cumulated sum.

The control limit depends on a number of variables:

- the desired target odds multiplier associated to an out-of-control process
- the false alarm probability α accepted
- number of simulations

Motivating example

To illustrate how **cusum** can be used for monitoring, we employ a simple and artificial data set generated to closely follow the performance data of German hospitals for one non-risk-adjusted performance indicator and one risk-adjusted performance indicator in 2016 and 2017.

risk-adj.	Indicator Description	Further explanation (in German)
NO	Ratio of observed to expected cases of severe stroke or death under open carotid stenosis surgery	pdf (p4)
YES	Preoperative stay more than 24 hours for patients with proximal femur fracture	pdf (p23)

Non-risk-adjusted performance indicator

```
data("cusum_example_data", package = "cusum")

head(cusum_example_data)
#>   t     y year
#> 1 1 FALSE 2016
#> 2 2 FALSE 2016
#> 3 3 FALSE 2016
#> 4 4 FALSE 2016
#> 5 5 FALSE 2016
```

```
#> 6 6 FALSE 2016
```

Risk-adjusted performance indicator

```
data("racusum_example_data", package = "cusum")

head(racusum_example_data)
#>   t     y   score year
#> 1 1 FALSE 0.00237 2016
#> 2 2 FALSE 0.00237 2016
#> 3 3 FALSE 0.02412 2016
#> 4 4 FALSE 0.01893 2016
#> 5 5 FALSE 0.00725 2016
#> 6 6 FALSE 0.00810 2016
```

First, CUSUM charts are constructed on performance data from 2016 (Phase I), and then applied and evaluated on performance data from 2017 (Phase II).

```
cusum_example_p1 <- cusum_example_data[cusum_example_data$year == 2016, ]
cusum_example_p2 <- cusum_example_data[cusum_example_data$year == 2017, ]

racusum_example_p1 <- racusum_example_data[racusum_example_data$year == 2016, ]
racusum_example_p2 <- racusum_example_data[racusum_example_data$year == 2017, ]
```

Non-risk-adjusted CUSUM chart

Simulation of CUSUM Control Limits

We get the control limit of our CUSUM chart by simulating for a false alarm probability depending on sample size and accepted failure probability.

We can estimate the accepted failure probability by taking the average of Phase I. Alternatively, we could also define an accepted failure probability politically

```
failure_probability <- mean(cusum_example_p1$y)

n_patients <- nrow(cusum_example_p1)
```

Then, control limits can be simulated using *cusum_limit_sim*.

```
cusum_limit <- cusum_limit_sim(failure_probability,
                               n_patients,
                               odds_multiplier = 2,
                               n_simulation = 1000,
                               alpha = 0.05,
                               seed = 2046)


print(cusum_limit)
#> [1] 6.498476
```

Applying CUSUM Charts

Monitoring via CUSUM charts is applied on performance data from 2017 (Phase II) and the control limit *cusum_limit*. It can be calculated using *cusum*.

```r
patient_outcomes <- cusum_example_p2$y


cusum_cs <- cusum(failure_probability,
                  patient_outcomes,
                  limit = cusum_limit,
                  odds_multiplier = 2,
                  reset = FALSE)

head(cusum_cs)
#>   t failure_probability ct signal    limit
#> 1 1               0.216  0      0 6.498476
#> 2 2               0.216  0      0 6.498476
#> 3 3               0.216  0      0 6.498476
#> 4 4               0.216  0      0 6.498476
#> 5 5               0.216  0      0 6.498476
#> 6 6               0.216  0      0 6.498476
plot(cusum_cs)
```

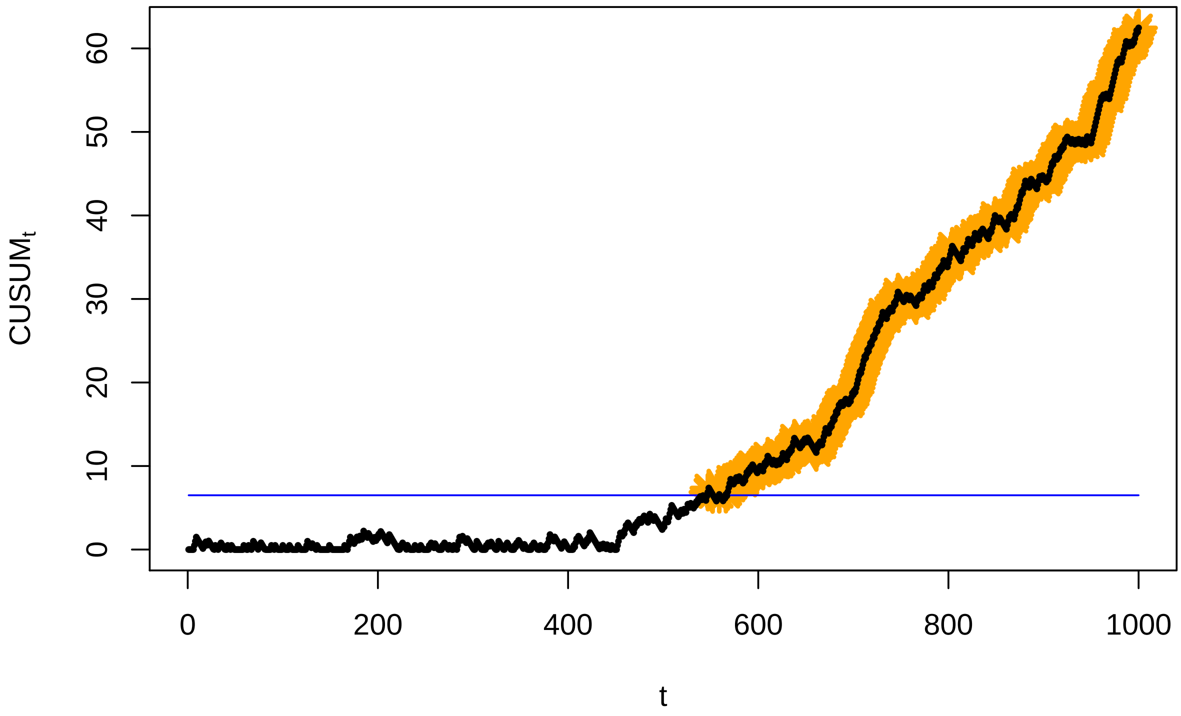

Performance is as expected during the first half of monitoring, and then deteriorates. We get a alarm at t=547. If reset==TRUE, the CUSUM resets after each alarm.

```r
cusum_cs <- cusum(failure_probability,
                  patient_outcomes,
                  limit = cusum_limit,
```

```
                    odds_multiplier = 2,
                    reset = TRUE)

plot(cusum_cs)
```

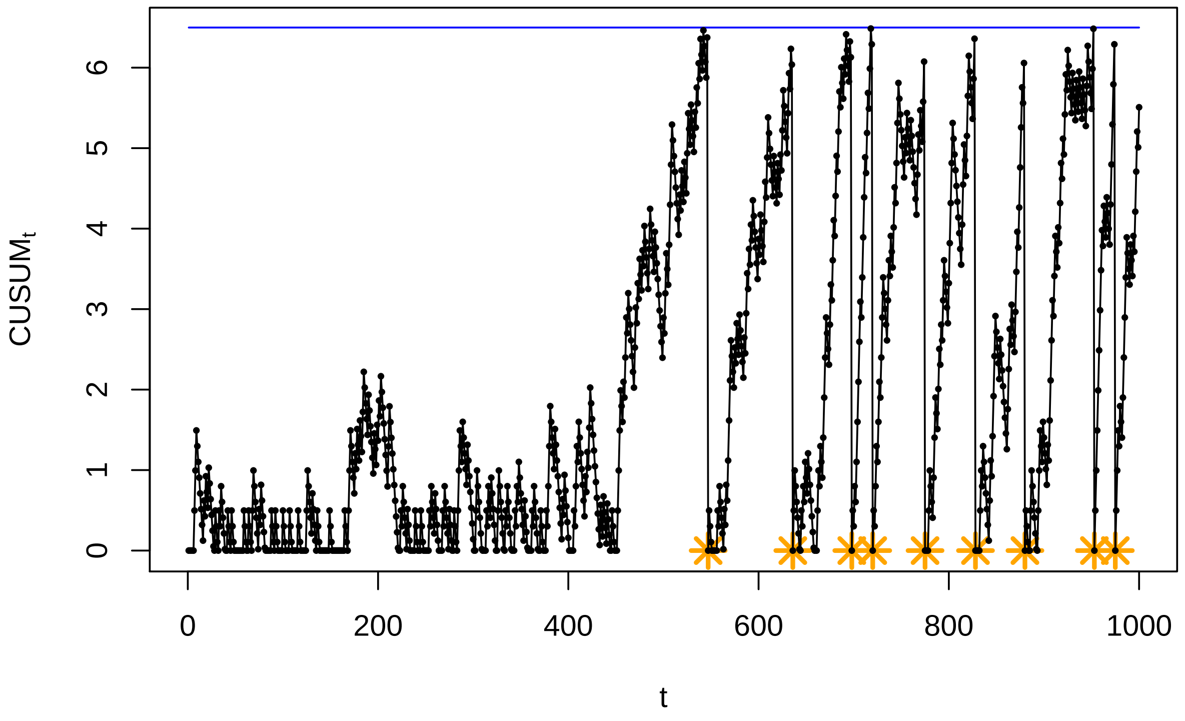

Evaluating CUSUM charts

The false alarm probability of a CUSUM chart can be simulated using *cusum_alpha_sim* given a predefined control limit.

```
n_patients <- nrow(cusum_example_p2)

cusum_alpha <- cusum_alpha_sim(failure_probability,
                               n_patients,
                               odds_multiplier = 2,
                               n_simulation = 1000,
                               limit = cusum_limit,
                               seed = 2046)

print(cusum_alpha)
#> [1] 0.05
```

We see that *cusum_alpha* equals our previously defined false alarm probability of 0.05.

Risk-adjusted CUSUM chart

Simulation of RA-CUSUM Control Limits

Control limits of RA-CUSUM charts are simulated for a false alarm probability depending on sample size and risk distribution.

RA-CUSUM Control limits can be simulated using *racusum_limit_alpha*.

```
patient_risks <- racusum_example_p1$score

racusum_limit <- racusum_limit_sim(patient_risks,
                                   odds_multiplier = 2,
                                   n_simulation = 1000,
                                   alpha = 0.05,
                                   seed = 2046)

print(racusum_limit)
#> [1] 3.742861
```

Applying RA-CUSUM charts

Monitoring via RA-CUSUM chart is applied on performance data from 2017 (Phase II) and the control limit *racusum_limit*. It can be calculated using *racusum*.

```
patient_risks <- racusum_example_p2$score

patient_outcomes <- racusum_example_p2$y

racusum_cs <- racusum(patient_risks,
                      patient_outcomes,
                      limit = racusum_limit,
                      odds_multiplier = 2,
                      reset = FALSE)
plot(racusum_cs)
```

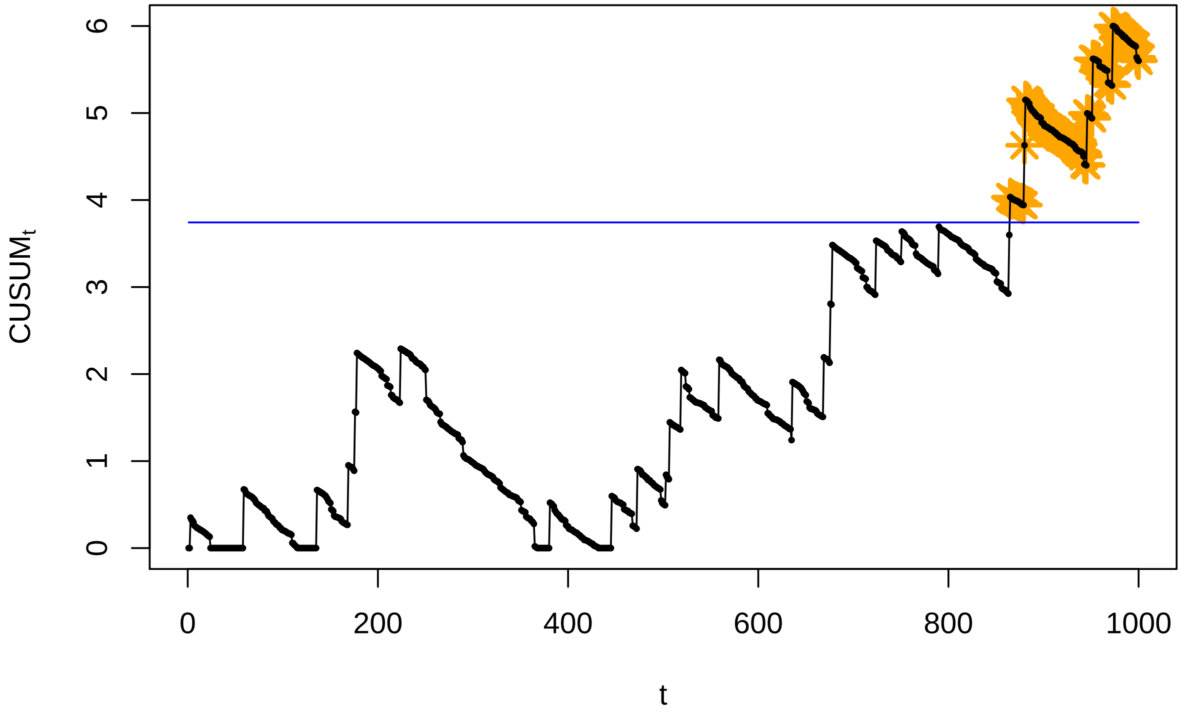

Performance is as expected during the first half of monitoring, and then deteriorates. We get a alarm at t=865. If reset==TRUE, the CUSUM resets after each alarm.

```
racusum_cs <- racusum(patient_risks,
                      patient_outcomes,
                      limit = racusum_limit,
                      odds_multiplier = 2,
                      reset = TRUE)

plot(racusum_cs)
```

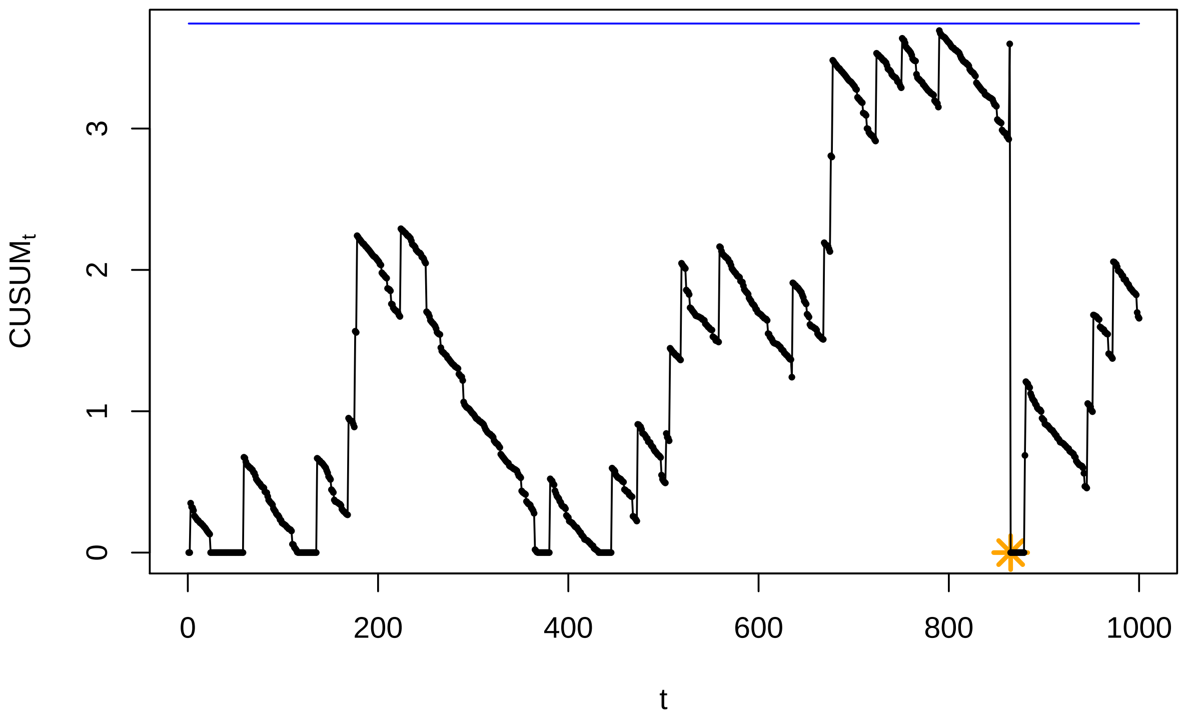

Evaluating RA-CUSUM charts

The false alarm probability of a CUSUM chart can be simulated using *cusum_alpha_sim*.

```
racusum_alpha <- racusum_alpha_sim(patient_risks,
                                   odds_multiplier = 2,
                                   n_simulation = 1000,
                                   limit = racusum_limit,
                                   seed = 2046)

print(racusum_alpha)
#> [1] 0.058
```

We see that *racusum_alpha* is similar to our previously defined false alarm probability of 0.05. Deviation is possible due to a slight change in risk population.

CUSUM Chart for process improvement

CUSUM charts for detecting process improvements can be constructed similarly, but the CUSUM statistic is restricted to non-positive values.

```
cusum_limit_improve <- cusum_limit_sim(failure_probability,
                                       n_patients,
                                       odds_multiplier = .5,
```

```
                                   n_simulation = 1000,
                                   alpha = 0.5,seed = 2046)

cusum_cs_improve <- cusum(failure_probability,
                          patient_outcomes = cusum_example_p2$y,
                          limit = cusum_limit_improve,
                          odds_multiplier = .5)

plot(cusum_cs_improve)
```

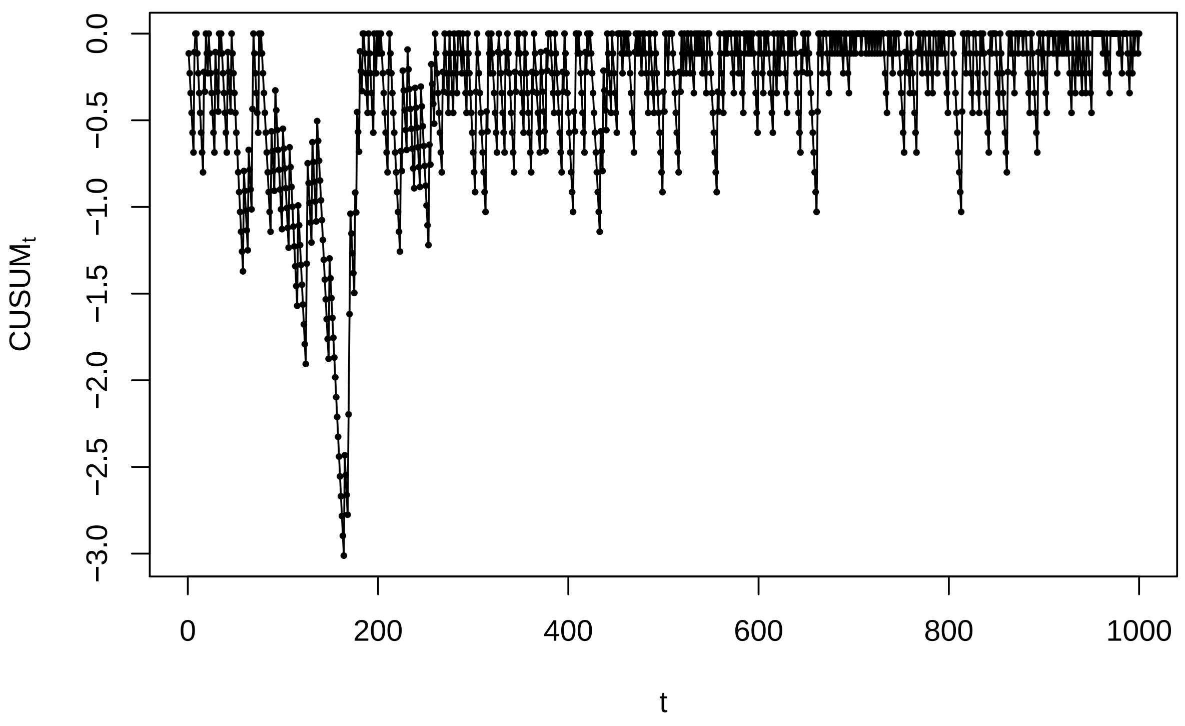


```
cusum_alpha_sim(failure_probability,
                n_patients,
                odds_multiplier = 0.5,
                n_simulation = 1000,
                limit = cusum_limit_improve,
                seed = 2046)
#> [1] 0.505
```
